# Peptide-Based Drug Development

**DOI:** 10.3390/biomedicines10082037

**Published:** 2022-08-21

**Authors:** William D. Lubell

**Affiliations:** Département de Chimie, Université de Montréal Complexe des Sciences, B-3015 1375 Avenue Thérèse-Lavoie-Roux, Montréal, QC H2V 0B3, Canada; william.lubell@umontreal.ca

The celebration of one hundred years of insulin therapy in 2021 marked a milestone for the application of peptide-based therapeutics [1]. Over eighty peptide drugs have since entered the market [2]. Bolstered by a higher rate of clinical trial success than small molecules [3], the ability to affect previously considered ‘undruggable’ targets such as protein–protein interactions [4], and a lower production cost compared to biologics [5], peptides now command an enviable position in the drug discovery and development process due in great part to high potency, keen specificity, and low toxicity [6]. The expansion of the field of peptide-based drug development will however necessitate overcoming challenges to optimize leads to generate clinical candidates that surmount limitations in bioavailability, metabolism by peptidases and proteases, as well as physicochemical and pharmacokinetic drawbacks [7].

Capturing a snapshot of this rapidly developing field, this issue on “Peptide-Based Drug Development” comprises seven original research contributions and three reviews which highlight various targets and methods currently under development. Sampling within a wide range of therapeutic indications, publications offer a good overview of peptide-based approaches including treatments for cancer, inflammation, chronic pain, as well as psychological and metabolic ailments. The relevance of cyclization and mimetic approaches for the enhancement of peptide properties such as bioavailability is well covered. Moreover, a glimpse of the future of peptide-based drug design is gleaned in a featured computational method for creating inhibitors of protein-protein interactions.

Towards therapy for treating chronic pain, the FDA approval of the peptide calcium channel blocker Ziconotide secured the importance of conotoxin venoms from predatory marine cone snails as a valuable source for drug discovery [8]. K.L. McMahon et al. now report the discovery, solution structure, and selective sodium channel inhibitory activity of a novel conotoxin, SxIIIC. Targeting the therapeutically relevant NaV1.7 subtype, SxIIIC proved to be among the most potent pore blockers. Displaying nanomolar potency, SxIIIC exhibited near irreversible inhibition of the NaV1.7 sodium channel subtype. In related research on the therapeutic potential of natural products from marine sources, two publications from O. Sintsova et al. describe, respectively, the neuroprotective and analgesic effects of peptides from sea anemone. The described peptides are relatively non-toxic members of the Kunitz-type serine protease inhibitor family. In one publication, certain peptides exhibited the ability to protect neuroblastoma Neuro-2a cells against the neurotoxin 6-hydroxydopamine. The neuroprotective activity was associated with the potential for reducing reactive oxygen species in neuronal cells. In a second publication, a different sea anemone peptide, HCRG21 was further pursued as an alternative to traditional analgesics due to its potential to inhibit the transient receptor potential vanilloid type 1 (TRPV1) cation channel. In models of mechanical and thermal hyperalgesia, HCRG21 demonstrated analgesic effects with similar potency and longer duration of action as the nonsteroidal anti-inflammatory drug indomethacin. The analgesic effects correlated with reduction of tumour necrosis factor-α levels as well as anti-inflammatory activity in a mouse hind paw edema assay.

To address the challenges of advancing peptide therapeutics, the development of methods for achieving improved bioavailability remains a significant goal [9]. D.G. Monteiro et al. explore the use of peptide head-to-tail cyclisation and selective amide *N*-methylation to develop bioavailable mimics of the peptide hormone hepcidin, which binds and inhibits the iron transport activity of the transmembrane protein ferroportin. In the interest of securing peptide-based drugs for lowering iron levels to treat pathologies such as β-thalassaemia and sickle cell disease, potent cyclic mini-hepcidin analogs were synthesized. Although the design gave high ferroportin binding affinity and improved serum stability, cyclisation and *N*-methylation strategies failed to improve membrane permeability. On the other hand, in a second publication on the theme of improving cyclic peptide bioavailability, N. P. Sturre et al. show the importance of a tryptophan residue for improving cell permeability. Targeting the growth factor receptor-bound protein 7 (Grb7) Src homology 2 (Grb-SH2) domain in an approach to breast cancer therapy, potent bicyclic peptides conjugated to the cell-penetrating peptide penetratin were previously shown to exhibit high affinity and activity in cells but low efficacy in animal models due likely to poor bioavailability. A novel Trp-bearing analog demonstrated comparable inhibitory activity in a wound healing assay of breast cancer cell migration in spite of having the lowest affinity for the Grb-SH2 domain.

In the search for modulators of emotional and behavioural functions such as arousal, appetite, anxiety, and sleep, peptide analogs have been actively pursued as agonists and antagonists of the Relaxin Family Peptide-3 (RXFP3) receptor [10]. Based on hydrocarbon cross-linked B-chain agonists, H.S. Lee et al. synthesized relaxin-3 peptide analogs possessing dithioether cross-links and α-aminoisobutyric acid (Aib) residues to stabilize the active α-helical conformer. Although cyclic dithioethers were less promising, judicious placements of Aib residues stabilized the α-helix in linear agonists. The study highlighted the relevance of threonine-21 for receptor engagement.

As mentioned, peptides offer new promise for blocking protein–protein interactions to regulate various biological processes. Although combinatorial approaches such as those using libraries from phage and mRNA display have proven remarkably effective for discovering potent peptide leads, new methods are desired for rationally designing protein–protein interaction blockers [4]. T. Kosugi and M. Ohue provide a valuable step towards such designs by adding a computational method to improve the solubility of de novo protein designed sequences.

Three review articles round out this thematic issue. In a pedagogic tour-de-force, a review on vasopressin is presented as the result of an edifying “write to learn” project conceived by Professor C. Gamberi featuring contributions from over seventy third-year Biology major undergraduate students. S. Sparapani et al. cover the historical, physiological, and pharmacological aspects of the antidiuretic peptide hormone. Surveying recent developments in preclinical and clinical research, the well written publication offers a useful focus on current developments of vasopressin agonists and antagonists to treat various cardiac and renal disorders, such as polycystic kidney disease. Professor Y.C. Boo reviews the application of peptide analogs, including melanocyte stimulating hormone derivatives, as modulators of melanin levels to treat skin pigmentary disorders. Peptide-based therapy for treating skin pigmentary disorders was validated when the α-melanocyte stimulating hormone analogue Scenesse entered the market for preventing skin damage from the sun in people with erythropoietic protoporphyria [11]. Highlighting the relevance of peptides in the melanin synthesis process, the review probes roles in tuning skin pigmentation and its potential for drug discovery. Finally, C. Proulx et al. review a peptide mimic approach with respect to the design of selective modulators of the cluster of differentiation 36 receptor (CD36). Linear and cyclic azapeptide modulators of CD36 were conceived to curb macrophage-driven inflammation and to mitigate atherosclerotic and angiogenic pathology. In mouse models, the modulators inhibited neovascularization, cardiovascular injury, and altered activated mononuclear phagocyte metabolism to decreased proinflammatory immune responses and alleviate subsequent inflammation-dependent neuronal injury associated with retinitis pigmentosa, diabetic retinopathy, and age-related macular degeneration.

Among the successful biomedicines in use today, peptides have established an important niche in the drug development spectrum complementing small molecule and biological therapeutics. Readily accessible by chemical and biological methods, peptides offer ideal properties for forming high affinity and specific interactions to target protein surfaces. Surveying peptide-based drug development in this thematic issue, many innovations are in sight on the horizon for meeting design and synthesis challenges to deliver bioavailable and metabolically stable prototypes with ideal pharmacological properties. A bright future of peptide-based drugs for improving quality of life awaits.
